# Spatial variability and changes of metabolite concentrations in the cortico‐spinal tract in multiple sclerosis using coronal CSI

**DOI:** 10.1002/hbm.22229

**Published:** 2012-12-26

**Authors:** Carmen Tur, Claudia A.M. Wheeler‐Kingshott, Daniel R. Altmann, David H. Miller, Alan J. Thompson, Olga Ciccarelli

**Affiliations:** ^1^ Department of Brain Repair and Rehabilitation UCL Institute of Neurology London United Kingdom; ^2^ Department of Medicine Clinical Neuroimmunology Unit Multiple Sclerosis Centre of Catalonia (CEM‐Cat) Autonomous University of Barcelona CARM‐Vall d'Hebron University Hospital Barcelona Spain; ^3^ Department of Neuroinflammation UCL Institute of Neurology London United Kingdom; ^4^ Medical Statistics Unit London School of Hygiene and Tropical Medicine London United Kingdom

**Keywords:** multiple sclerosis, MRI, MRS

## Abstract

We characterized metabolic changes along the cortico‐spinal tract (CST) in multiple sclerosis (MS) patients using a novel application of chemical shift imaging (CSI) and considering the spatial variation of metabolite levels. Thirteen relapsing‐remitting (RR) and 13 primary‐progressive (PP) MS patients and 16 controls underwent ^1^H‐MR CSI, which was applied to coronal‐oblique scans to sample the entire CST. The concentrations of the main metabolites, i.e., *N*‐acetyl‐aspartate, myo‐Inositol (Ins), choline containing compounds (Cho) and creatine and phosphocreatine (Cr), were calculated within voxels placed in regions where the CST is located, from cerebral peduncle to corona radiata. Differences in metabolite concentrations between groups and associations between metabolite concentrations and disability were investigated, allowing for the spatial variability of metabolite concentrations in the statistical model. RRMS patients showed higher CST Cho concentration than controls, and higher CST Ins concentration than PPMS, suggesting greater inflammation and glial proliferation in the RR than in the PP course. In RRMS, a significant, albeit modest, association between greater Ins concentration and greater disability suggested that gliosis may be relevant to disability. In PPMS, lower CST Cho and Cr concentrations correlated with greater disability, suggesting that in the progressive stage of the disease, inflammation declines and energy metabolism reduces. Attention to the spatial variation of metabolite concentrations made it possible to detect in patients a greater increase in Cr concentration towards the superior voxels as compared to controls and a stronger association between Cho and disability, suggesting that this step improves our ability to identify clinically relevant metabolic changes. Hum Brain Mapp 35:993–1003, 2014. © 2012 The Authors. Human Brain Mapping Published by Wiley Periodicals, Inc.

## INTRODUCTION

An increasing number of studies using quantitative magnetic resonance (MR) techniques have focused on detecting damage in the normal‐appearing brain tissue [Chard et al., 2002; Khaleeli et al., 2008, 2007; Sastre‐Garriga et al., 2005; Tiberio et al., 2006; Tur et al., 2011] with the ultimate goal of understanding the mechanisms accounting for the development of irreversible disability in multiple sclerosis (MS). ^1^H‐MR spectroscopy (MRS) is amongst the most pathologically specific techniques, since it allows in vivo quantification of the concentrations of the most abundant metabolites [Chard et al., 2002; Sastre‐Garriga et al., 2005; Tiberio et al., 2006], which reflect the underlying pathological processes. In particular, MRS allows quantification not only of *N*‐acetyl‐aspartate (NAA), which is a marker of neuronal health and integrity [Moffett et al., 2007], but also of other important metabolites, such as *myo‐*inositol (Ins) and choline (Cho), which reflect glial proliferation (and activation) and membrane turnover associated with inflammation [Bitsch et al., 1999], respectively. Abnormal concentrations of metabolites, including NAA and Ins, have been reported in the brain [Chard et al., 2002; Sastre‐Garriga et al., 2005; Tiberio et al., 2006] and spinal cord [Ciccarelli et al., 2007; Marliani et al., 2010, 2007] of patients with MS when compared with controls, and are associated with motor disability [Chard et al., 2002; Sastre‐Garriga et al., 2005] and cognitive impairment [Summers et al., 2008].

Many spectroscopy studies have investigated relatively large volumes in the central nervous system (CNS) using chemical‐shift imaging (CSI), which permits excitation of a large spectroscopic volume located on axial slices and collects the spectral data from many voxels [Chard et al., 2002; Sastre‐Garriga et al., 2005]. The reported metabolite concentrations, therefore, represent an “averaged” concentration over different CNS locations and tissues [Barker et al., 2006]. On the other hand, single‐voxel MRS has been successfully used to quantify metabolites at specific locations in the brain white matter (WM) [Fernando et al., 2004] and gray matter (GM) [Geurts et al., 2004], providing little information on the spatial distribution of metabolites.

Here, we localized CSI on a coronal slice passing through the cortico‐spinal tract (CST) in order to investigate metabolic changes along the entire motor pathway, from the internal capsule to the corona radiata in patients with different types of MS within a single acquisition protocol. We hypothesized that patients with primary‐progressive MS (PPMS) would show lower NAA and Cho than relapsing‐remitting MS (RRMS), suggesting greater axonal damage [Tallantyre et al., 2009] and less inflammatory demyelination [Bramow et al., 2010]. To understand whether metabolic changes were clinically relevant, the associations between metabolite concentrations and disability were investigated in each patient group. This study builds on previous work, which has demonstrated that CST structural abnormalities detected by MRI have a direct impact on the motor deficits observed in patients with MS [Gorgoraptis et al., 2010; Lee et al., 2000; Reich et al., 2007]. Since it has been recently suggested that the ability to detect pathological differences between MS patients and healthy controls may be significantly increased by accounting for spatial variation of imaging measures from one end of a tract to the other [Goldsmith et al., 2011], we have repeated our analysis by taking into account the variability of metabolite concentrations that is attributed to the specific location of the voxel within the CST.

## METHODS

### Subjects and Clinical Assessment

Thirteen consecutive patients with RRMS [Lublin and Reingold, 1996], 13 patients with PPMS [Thompson et al., 2000], and 16 healthy controls were invited to participate in this study, which was approved by the Ethics Committee of the National Hospital for Neurology and Neurosurgery and UCL Institute of Neurology; all participants gave written informed consent.

All patients were scored on the expanded disability status scale (EDSS) [Kurtzke, 1983], timed walk test (TWT) and nine‐hole peg test (NHPT) [Cutter et al., 1999]. Those patients who were not able to walk were assigned a TWT score of 180 s and those patients who were not able to complete the NHPT were assigned a NHPT score of 300 s [Hoogervorst et al., 2002].

### MRI Protocol

**Figure 1:**
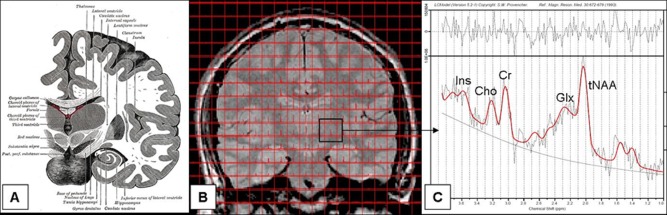
Chemical shift imaging grid covering the entire CST on a single image shown together with a schematic picture of the anatomy of the CST and a spectrum. (**A**) Coronal brain image, from the 20th U.S. edition of Gray's Anatomy of the Human Body (20th edition, thoroughly revised and re‐edited by Warren H. Lewis, Illustrated with 1247 engravings. Philadelphia: Lea & Febiger, 1918, published in 1918); (**B**) CSI grid covering the entire CST on a single (FSE) image. This grid shows the original location of voxels; (**C**) Spectrum from the voxel located above the left cerebral peduncle in a healthy control. CSI, Chemical shift imaging; tNAA, *N*‐acetylaspartate and *N*‐acetlylaspartylglutamate; Glx, glutamate plus glutamine (data not reported); Cr, creatine and phosphocreatine; Cho, choline‐containing compounds; Ins, myo‐inositol.

All subjects underwent the following MR protocol on a GE 1.5T scanner:

(i) T1‐weighted 3D FSPGR (coronal‐oblique) imaging (TE =5.104 ms, TR = 14.3 ms, TI (inversion time) = 450 ms, NEX = 1, image dimensions = 128 × 256, voxel size = 1.2 × 1.2 × 1.2 mm^3^).
(ii) Dual‐echo fast spin echo (FSE) axial and coronal‐oblique imaging (TE = 17 ms, TR = 2500, NEX = 1, number of echoes = 2, voxel dimensions = 0.9 × 0.9 × 5 mm^3^). The coronal‐oblique images in (ii) and (iii) were acquired perpendicularly to the main axis (i.e., top) of the corpus callosum. In this way, the chemical shift imaging (CSI) grid (see Fig. 1) covered the entire CST with a single 2D spectroscopy protocol [see (iii)].
(iii) ^1^H‐MR volume‐selective CSI 2D sequence with a point resolved spectroscopy localization scheme (PRESS), to acquire data from a volume located on a coronal‐oblique scan to image the CST along its main axis (Fig. 1) (TE = 30 ms, TR = 3000 ms, number of excitations =1, section thickness = 15 mm, 24 × 24 phase encoding steps over a field of view of 220 × 220 mm, 512 points, nominal single voxel volume of 1.26 ml). To optimize the magnetic field homogeneity, local shimming was performed, using the water signal in the absence of any suppression. Afterwards, to achieve water suppression, a chemical shift selective saturation pulse (CHESS) was used. Finally, the unwanted signal from lipids was eliminated by means of outer volume saturation bands, positioned adjacent to the edges of the PRESS‐selected volume. Throughout the study, we performed a quality assurance (QA) programme that involved a phantom containing known concentration of NAA (50 mmol), using a single voxel PRESS protocol, and the “Braino” phantom (GE Medical Systems, Milwaukee, WI), containing 12.5 m*M* of NAA, 10 m*M* of Cr, 3 m*M* of Ch, 7.5 m*M* of ml, 12.5 m*M* of Glu, 5 m*M* of Lac, sodium azide (0.1%), 50 m*M* of potassium phosphate monobasic (KH_2_PO_4_), 56 m*M* of sodium hydroxide (NaOH) and 1 ml/l of Gd‐DPTA (Magnevist), using the CSI PRESS protocol. This QA routine allowed us to confirm the stability of the measurements and to obtain the calibration factor, which was then used in the LCModel analysis for metabolite quantification.

### Image Analysis

Lesions were contoured on the 13 T1‐weighted images that corresponded to the spectroscopic grid, using the FSE images as reference, with DispImage (D.L. Plummer, University College of London, London, UK) [Plummer, 1992; Sailer et al., 1999]. T1‐images were then segmented into WM, GM, cerebrospinal fluid (CSF), and lesions, using SPM8 (Functional Imaging Laboratory, Wellcome Department of Imaging Neuroscience, London).

**Figure 2:**
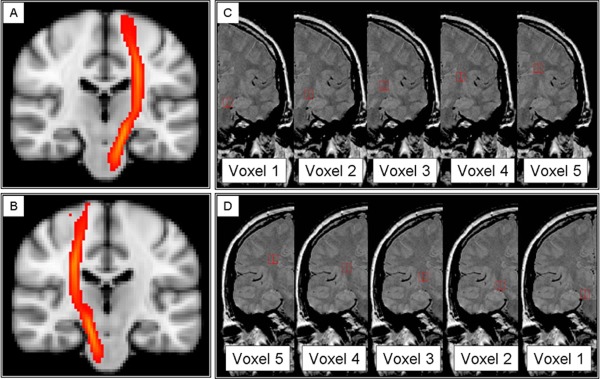
Voxels extracted from the spectroscopic grid to obtain concentrations of metabolites along the cortico‐spinal tract. (**A** and **B**) Probability map of the left and right cortico‐spinal tracts, provided by the JHU white matter tractography atlas (http://www.fmrib.ox.ac.uk/fsl), which were used, together with the individual T1 images, as reference images to identify and position the voxels along the CST. (**C** and **D**) Spectroscopic voxels (in white, 1 = cerebral peduncle, 2 = above cerebral peduncle, 3 = internal capsule, 4 = above internal capsule, 5 = corona radiata) selected in the left (C) and right (D) white matter in regions where the CST is known to be located, from the cerebral peduncle to the corona radiata, overlaid onto coronal PD images.

MRS imaging data were processed using SAGE–IDL 2005.3 (GE, Milwaukee). First, five voxels were selected and placed by an experienced observer (C.T.) on each side of the brain, one above the other, without overlapping, in five regions of the white matter where the CST is known to be located [i.e., cerebral peduncle, above the cerebral peduncle, internal capsule, above the internal capsule, corona radiata (Fig. 2)]. This step was performed using the anatomical landmarks visible on the coronal proton‐density images and with reference to both the individual T1‐weighted images and the JHU tractography‐atlas (http://www.fmrib.ox.ac.uk/fsl/), which was used to display the CST (Fig. 2). Secondly, voxels were extracted from the spectroscopic grid, one at a time, and the segmented WM, GM, CSF and lesional tissue within each voxel, were obtained. A fraction for each segment was then calculated and retained to be used as covariates in the statistical analysis. Finally, using the LCModel (*Version 5.2‐1*) [Provencher, 1993], metabolite quantification in mmol/l was obtained within the extracted voxels. In particular, the concentrations of NAA plus *N*‐acetyl‐aspartyl‐glutamate (tNAA), choline‐containing compounds (Cho), creatine and phosphocreatine (Cr), and myo‐inositol (Ins) were obtained and retained only if their associated SD was <20%.

The number of voxels used in the following steps of the analysis was 398 for tNAA (151, 121, and 126 for controls, RRMS and PPMS, respectively); 353 for Cho (129, 108, and 116, for controls, RRMS and PPMS, respectively); 356 for Cr (132, 106, and 118, for controls, RRMS and PPMS, respectively); 213 for Ins (73, 64, and 76, respectively).

### Statistical Analysis

In the patient and control group, we tested for differences in metabolite concentrations between right and left voxels using the Wilcoxon's test.

### Differences Between Groups

Linear mixed regression models were used to detect differences in the metabolite concentrations between (i) RRMS and PPMS patients, (ii) each patient group and controls. The metabolite concentration was used as the dependent variable, and a variable indicating the subject group as explanatory variable, adjusting for age, gender, WM, CSF, and lesion fractions, and side (e.g., left or right). The variables that significantly contributed to explaining the variance in the dependent variable were retained in the final model. For each metabolite, all the available voxels were examined at the same time. This was a conservative statistical approach, which reduced the risk of obtaining sporadic results and false positives.

To investigate whether attention to the spatial variation of the metabolite concentrations along the CST improved the ability of the statistical model to detect differences between groups, the analysis was repeated by including a variable indicating the position of the voxel; whenever there was evidence of a linear effect of position, this variable was kept in the model; otherwise, we included in the model a set of five indicators of position, one for each voxel. To investigate the effect of the spatial variation on the differences in metabolite levels between groups, we repeated the analysis by also modeling the interaction between group and voxel location.

### Association Between Metabolite Concentrations and Disability

To investigate the association between metabolite concentrations and clinical scores in patients, the linear mixed regression models described above were used with the addition of an explanatory variable indicating the level of disability. For the EDSS, an indicator of two EDSS categories [e.g., EDSS below and above (or equal to) 4.5] was included. The 4.5 cut‐off for the EDSS was chosen based on the median EDSS for the whole group (Table 1). For the other clinical variables, we used the inverse values of TWT and NHPT (given their non‐normal distribution), which were entered, in turn, as independent variables. To assess whether attention for the spatial variation of metabolite levels improved our ability to detect associations between metabolite concentrations and clinical measures, the analysis was repeated by including the variable indicating the position of the voxel.

**Table 1:** Clinical and demographical characteristics of the subjects

	Patients			RRMS vs. PPMS	Controls	Patients vs. controls
	All patients	RRMS^a^	PPMS^a^	*P*		*P*
Age (yr)						
mean	49.615	45.615	53.615	<0.001^b^	40.375	<0.001^b^
SD (range)	12.189 (21, 67)	11.704 (21, 67)	11.361 (30, 65)		12.856 (26, 65)	
EDSS						
median (range)	4.240 (2, 7.500)	4 (2, 6)	5 (2.500, 7.500)	<0.001^c^	–	–
TWT (s)						
mean	22.685	7.710	36.508	<0.001^b^	–	–
SD (range)	46.771 (4.760, 180)	2.245 (4.9, 12)	61.779 (4.760, 180)			
HPT (s)						
mean	36.772	35.540	37.910	0.310^b^	–	–
SD (range)	37.548 (18.870, 162.710)	37.510 (19.590, 158.570)	37.690 (18.870, 62.710)			
Disease duration (yr)						
mean	10.160	7.770	12.750	<0.001^b^	–	–
SD (range)	7.820 (1, 27)	7.700 (1, 25)	7.110 (3, 27)			

a All the RRMS, but none of the PPMS patients, were on disease modifying treatment. None of the patients had received corticosteroid treatment over the three months prior to the study.

b *t*‐test

c Two‐sample Wilcoxon rank‐sum (Mann–Whitney) test.

## RESULTS

No significant differences were found between the left and right voxels, for all groups across all metabolite concentrations.

### Differences Between Groups

As expected, patients with PPMS were older, more disabled, especially in their ability to walk, and with a longer disease duration, than patients with RRMS (Table 1). The WM was the main tissue within the spectroscopic voxels (the mean fraction of WM across all voxels was of 87.5% in patients and 91.1% in controls). In patients, the mean fractions of GM and lesional tissue were 5.3% and 3.5%, respectively.

**Figure 3:**
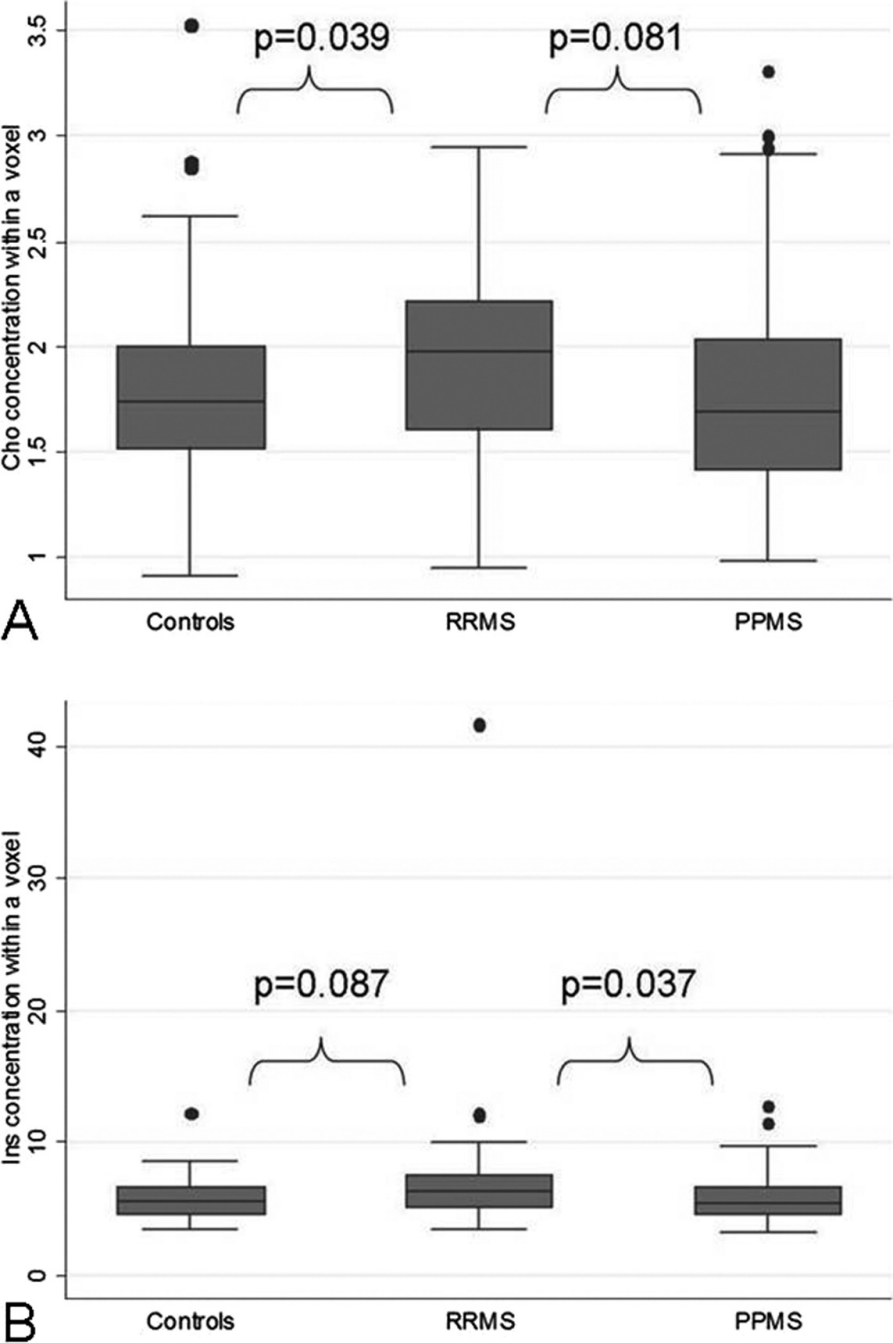
Differences in Cho and Ins between patients and controls. (**A**) A significantly lower concentration (in mmol/l) of Cho was seen in controls when compared to RRMS, and (**B**) lower Ins (in mmol/l) was seen in PPMS when compared to RRMS. RRMS, relapsing‐remitting multiple sclerosis; PPMS, primary progressive multiple sclerosis; Cho, Choline containing compounds (Cho); Ins, Myo‐Inositol.

After correcting for age, gender, voxel tissue contents, and side, RRMS patients showed a significantly higher Cho concentration in the CST than healthy controls (*P* = 0.039) and a trend towards a higher Cho concentration than PPMS patients (*P* = 0.081) (Table 2 and Fig. 3); they also showed a significantly higher CST Ins concentration than PPMS (*P* = 0.037) and a trend towards a higher CST Ins concentration than controls (*P* = 0.087) (Table 2 and Fig. 3). No other significant differences between groups in metabolite concentrations were observed.

**Table 2:** Metabolite concentrations and differences between groups

Metabolites	Mean (SD) mmol/l^a^		
	Controls	RRMS	PPMS
tNAA	10.658 (1.186)	10.286 (0.746)	10.105 (0.919)
Cho	1.793 (0.199)	1.938 (0.136)^b^	1.762 (0.313)
Cr	6.080 (0.387)	6.294 (0.626)	6.054 (1.076)
Ins	5.864 (0.809)	8.142 (5.734)^c^	5.711 (0.896)

tNAA, *N*‐acetylaspartate and *N*‐acetlylaspartylglutamate; Cho, choline‐containing compounds; Cr, creatine and phosphocreatine; Ins, myo‐inositol.

a This table shows the mean value (and SD) of each metabolite concentration for each group of subjects. Each subject contributed to mean of the appropriate group with one number, representing the mean value of each metabolite concentration within each subject, regardless of voxel position along the CST; note that these values do not correspond to the tNAA mean values used in the statistical analysis, which were the individual values per subject per position.

b Difference between RRMS and controls: *P* = 0.039.

c Difference between RRMS and PPMS *P* = 0.037 (after adjusting for age, gender, and voxel tissue contents).

**Figure 4:**
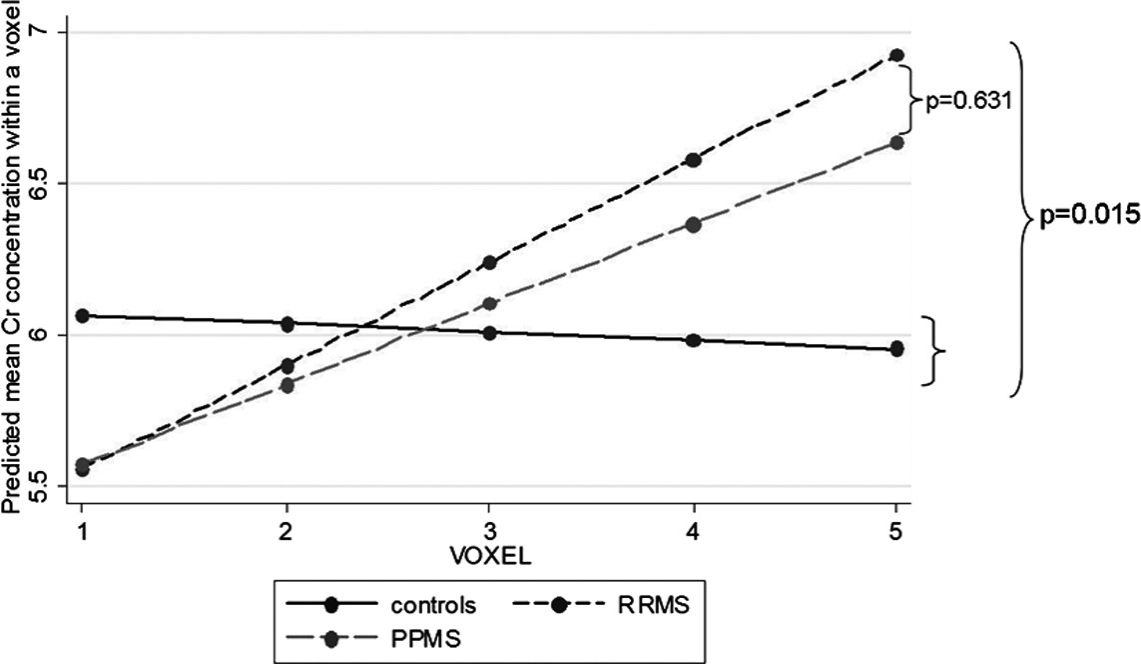
Differences between patients and controls in the slope of Cr along the CST. Both groups of patients showed a significant increase of Cr concentration (in mmol/l) towards the upper parts of the CST (for RRMS: *P* = 0.007; for PPMS: *P* = 0.017), whereas controls did not show such increase (*P* = 0.791). The slope of Cr significantly differed between patients and controls (*P* = 0.015), whilst it did not change between RRMS and PPMS (*P* = 0.631).

**Figure 5:**
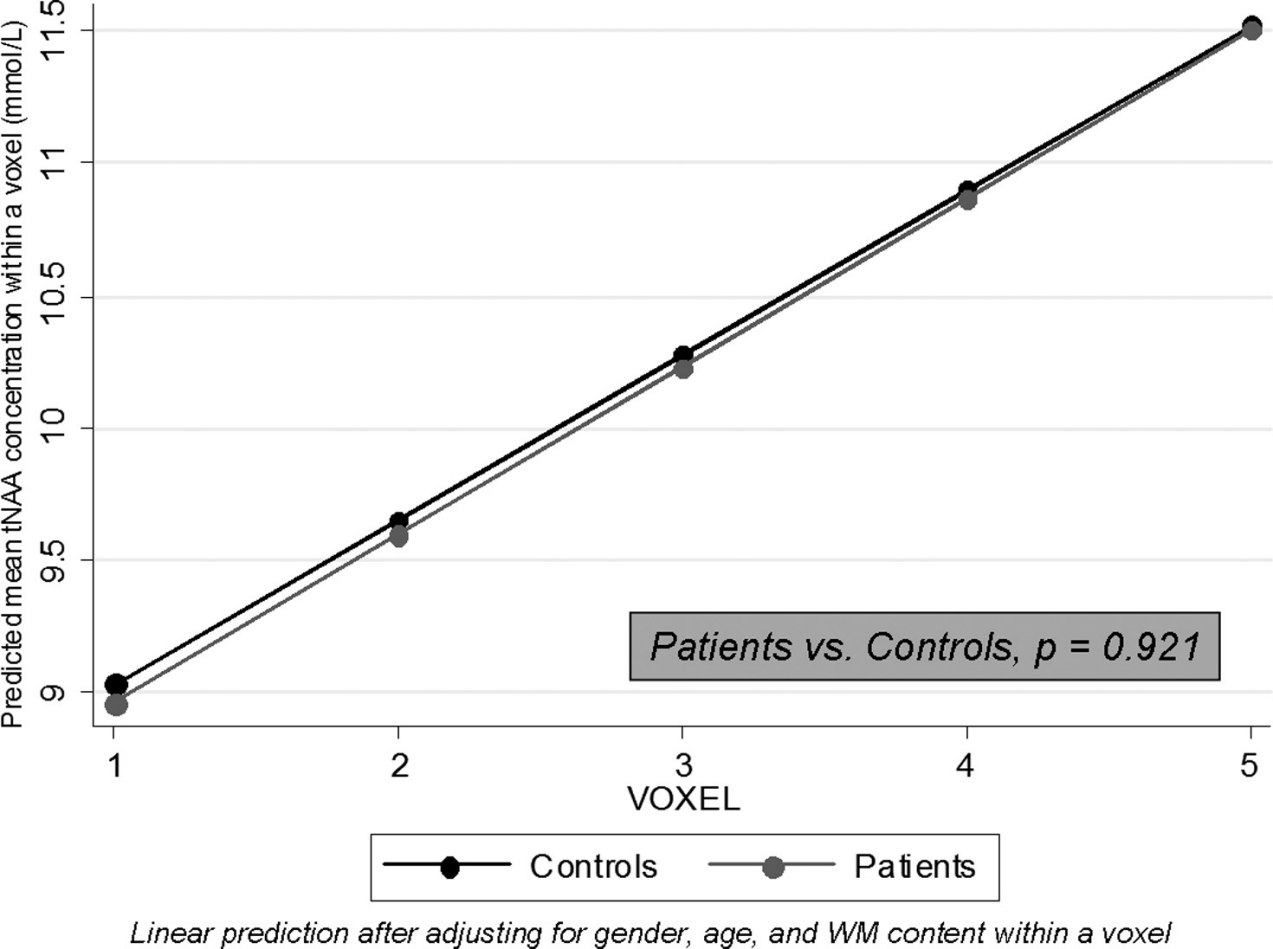
Predicted tNAA concentrations within a voxel. Both patients and controls showed a significant increase of tNAA concentration (in mmol/l) towards the upper parts of the CST after adjusting for age, gender, and WM content within a voxel (patients: *P* < 0.001; controls: *P* < 0.001). However, this increase was not significantly different between patients and controls. Voxel location: 1 = cerebral peduncle, 2 = above cerebral peduncle, 3 = internal capsule, 4 = above internal capsule, 5 = corona radiata. CI, confidence interval; CST, cortico‐spinal tract; RC, regression coefficient; t‐NAA, *N*‐acetyl‐aspartate plus *N*‐acetyl‐aspartyl‐glutamate; WM, white matter.

When spatial variation was taken into consideration, differences in Cr concentrations between patients and controls became significant, independently from GM, WM, and lesion fractions within voxels. Both RRMS and PPMS patients showed a significant (linear) increase in Cr concentration from the cerebral peduncle to the corona radiata: in RRMS, there was a mean increase per voxel proximity to corona radiata of 0.341 mmol/l [95% Confidence interval (95% CI) 0.095, 0.588], *P* = 0.007; in PPMS, there was a mean increase per voxel proximity to corona radiata of 0.265 [(95%CI) 0.048, 0.482], *P* = 0.017. Instead, this increase in Cr concentration was not seen in controls (Fig. 4). In fact, all patients together showed a greater increase in Cr concentration towards the voxels positioned superiorly compared with controls (Interaction coef. (95% CI) 0.32 (0.06, 0.60), *P* = 0.015) (Fig. 4). All patients together showed a mean increase in tNAA concentration along the CST from the inferior regions to the corona radiate of 0.634 mmol/l [(95% CI) 0.462, 0.805] *P* < 0.001; controls showed an increase of 0.619 mmol/l [(95% CI) 0.381, 0.858], *P* < 0.001 (Fig. 5); this increase in tNAA concentration was not significantly different between groups (*P* = 0.921), and was significant even when correcting for WM fraction change across the CST, which, in turn, showed, in all subjects together, a significant increase towards the voxels located in the corona radiata of 0.031 [(95% CI) 0.021, 0.042] *P* < 0.001. The other metabolites did not show a significant spatial variation along the CST and the differences in their concentration between groups were not affected by accounting for their spatial variation.

### Associations Between Metabolite Concentrations and Disability

**Figure 6:**
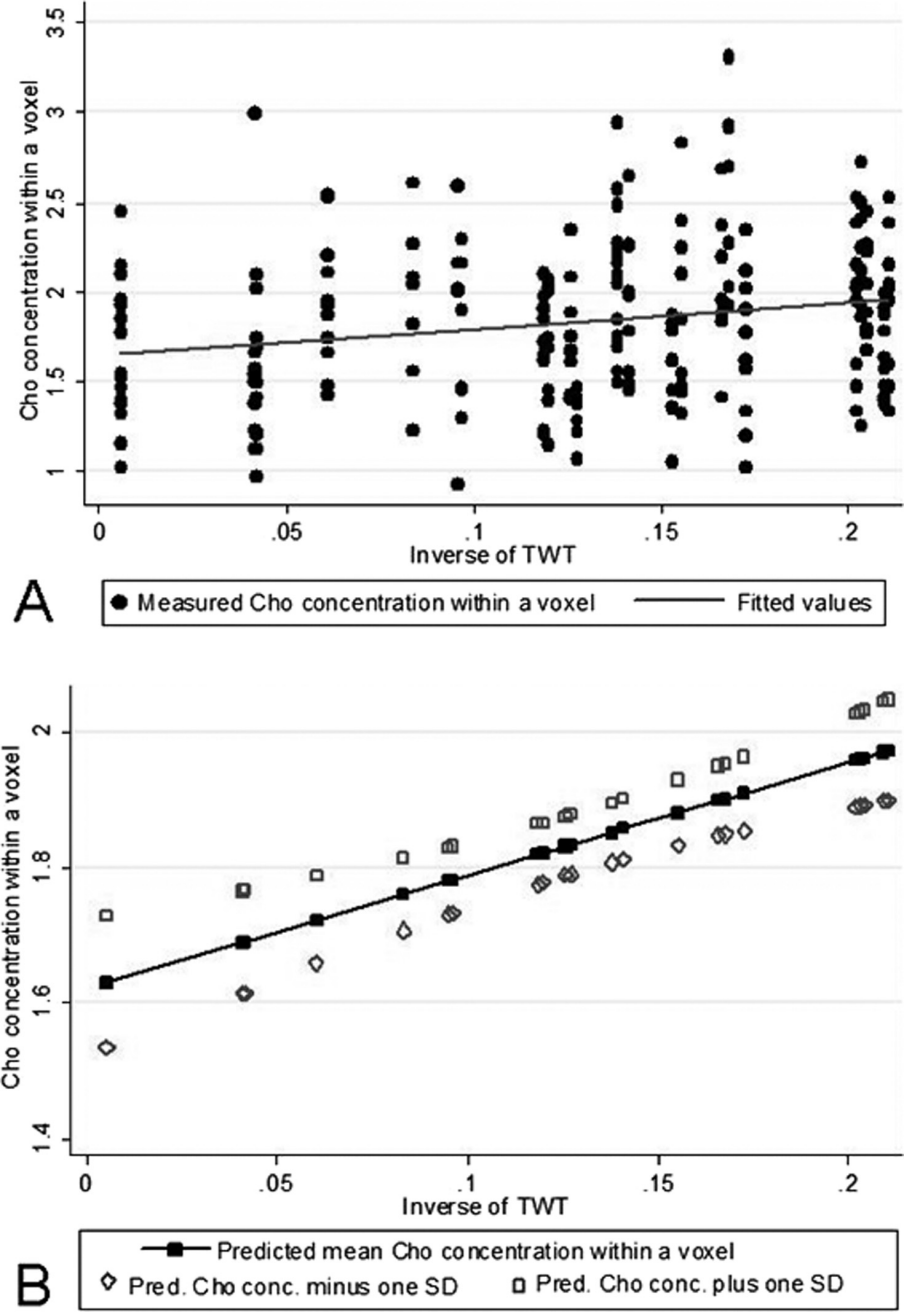
Association between Cho concentration in the CST and ability to walk before and after adjusting for the spatial variability of Cho concentrations along the CST. Patients with reduced ability to walk (as measured byt the inverse of TWT) showed lower concentrations of Cho (in mmol/l) in the CST (*P* = 0.019); (**A**) shows this relationship using the raw, unadjusted concentrations of Cho; (**B**) shows this relationship using the predicted mean Cho concentrations (± one SD) after adjusting for age, gender, tissue fractions, and spatial variation (voxel location and side).

In the whole group of patients, lower CST Cho concentrations were associated with worse walking ability, as measured by the TWT scores. Thus, there was a mean increase in Cho concentration of 1.538 mmol/l [Regression coefficient, RC (95% CI) 1.538 (0.064, 3.011), *P* = 0.041] per unit of higher *inverse TWT* (in 1/s). When spatial variation was taken into account, the association between TWT scores and Cho concentrations became slightly stronger: there was a mean increase in Cho concentration of 1.664 mmol/l [RC (95% CI) 1.664 (0.274, 3.054), *P* = 0.019], per unit of higher *inverse TWT* (in 1/s) (Fig. 6).

**Figure 7:**
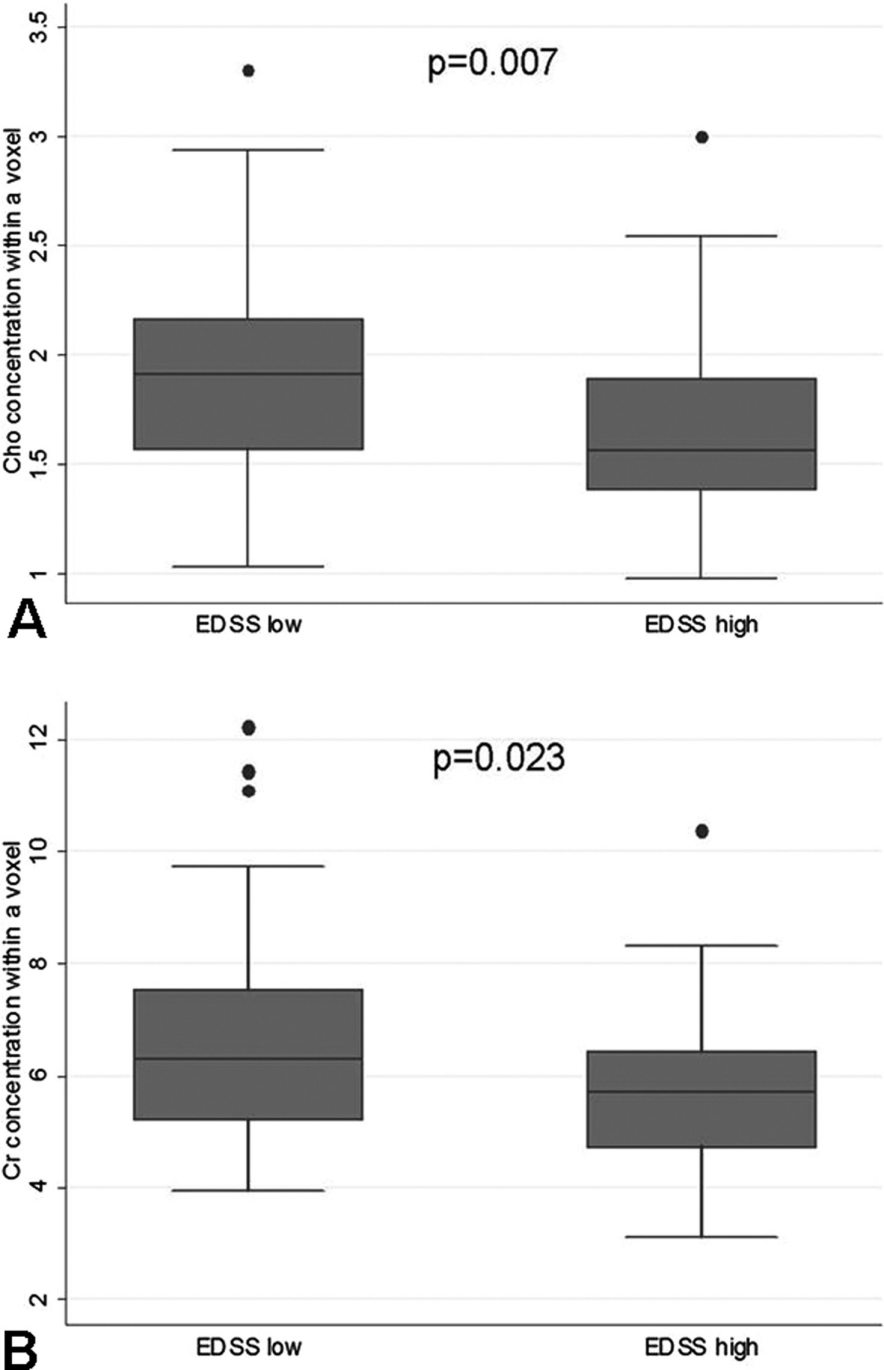
Association between metabolite concentrations and disability. PPMS patients with higher disability (as measured by the EDSS) showed lower concentrations of Cho (**A**) and Cr (**B**) in the CST (see text for more details on these relationships). Here, values of Cr and Cho concentrations refer to those obtained considering all CST voxels (left and right). All concentrations are expressed in mmol/l.

In the PPMS group, lower concentrations of Cho and Cr in the CST were associated with higher EDSS: per each EDSS point increase there was a decrease in Cho concentration of 0.348 mmol/l [RC (95% CI) −0.348 (−0.602, −0.094), *P* = 0.007], and a decrease in Cr concentration of 0.990 mmol/l [RC (95% CI) −0.990 (−1.841, −0.138), *P* = 0.023] (Fig. 7A,B). When spatial variation was taken into account, no major improvements in the strength of the associations were observed for Cho [RC (95% CI) −0.362 (−0.609, −0.115), *P* = 0.004] or Cr [RC (95% CI) −0.806 (−1.595, −0.0163), *P* = 0.045].

In RRMS patients, higher Ins concentrations were associated with worse walking performance, as measured by TWT: there was an increase in Ins concentration of 17.08 mmol/l per unit of lower *inverse TWT* (in 1/s) [RC (95% CI) −17.08 (−31.95, −2.22), *P* = 0.024]. No improvement in this association was observed when spatial variation was taken into account.

## DISCUSSION

We used coronal‐oblique CSI to sample metabolite concentrations along voxels placed in the WM where the CST is known to be located, and found that metabolic abnormalities occurred in patients with MS when compared with healthy controls. More specifically, we found a significantly higher Cho concentration in RRMS than in controls, who showed similar Cho concentrations to PPMS. We also found a significantly higher Ins concentration in RRMS than in PPMS, who showed a similar concentration to controls. When considering the pathological processes that have been associated with an increase in Cho and Ins [Bitsch et al., 1999; Kirov et al., 2009; Pan et al., 1996], these findings would be compatible with more extensive inflammation with increased membrane turnover [Frischer et al., 2009; Kirov et al., 2009], and greater glial hyperplasia and proliferation in the CST of patients with RRMS. These results extend previous investigations, which have reported increased levels of Ins in all disease types, including RR [Chard et al., 2002; Geurts et al., 2004; Marliani et al., 2010; Vrenken et al., 2005] and PPMS [Geurts et al., 2006; Sastre‐Garriga et al., 2005; Vrenken et al., 2005], by directly comparing these two clinical types of MS, and by focusing the investigation on the CST, which mediates motor function, and its damage has been reported to be crucial for the development of disability in MS [Bodini et al., 2009; Giorgio et al., 2010; Gorgoraptis et al., 2010; Pagani et al., 2005; Tallantyre et al., 2009].

An interesting finding of our study is that attention for spatial variation of the metabolite concentrations improved the detection of differences in metabolite levels between patients and controls. In particular, when taking into account the spatial variation in the statistical models, patients showed a greater increase in Cr concentration towards the superior parts of the CST as compared with controls. This steady and linear increase in Cr levels observed in patients could be interpreted as a metabolic response to structural tissue damage occurring in the CST [Gorgoraptis et al., 2010; Lee et al., 2000; Reich et al., 2007]. Increased Cr (e.g., the sum of phosphocreatine and creatine) may reflect increased energy metabolism [Hajek and Dezortova, 2008], which is especially requested in the more superior regions of the white matter along the CST. The main message coming from this finding is that a comprehensive assessment of metabolic damage in patients with MS has to pay attention to the spatial variation of the metabolite concentrations along a tract, which may improve the ability to distinguish pathological from healthy tissue using imaging.

When we investigated the associations between metabolite levels and disability, we found that, in the whole group of patients, a lower Cho concentration was associated with worse walking ability, as measured by the TWT. In the PPMS group, a lower Cho concentration correlated with greater disability, as measured by the EDSS. These associations suggest that membrane turnover associated with inflammation, as reflected by Cho concentration, may be lower in patients with greater disability. Since we included the amount of lesional tissue within a voxel as a covariate in all our analyses, it is unlikely that the presence of fewer lesions in the PPMS group has significantly contributed to this finding. This is in agreement with recent neuropathological evidence for less pronounced inflammation in the normal‐appearing WM in the later phases of the progressive stage of the disease, where the inflammatory infiltrates of T, B, and plasmatic cells seem to be reduced when compared with those seen in earlier stages of the disease [Frischer et al., 2009]. However, extensive microglia activation, behind a closed or repaired blood brain barrier, has been described in progressive MS cases [Frischer et al., 2009; Moll et al., 2011]. It is known that activated microglia show changes in both morphology and expression of cell surface antigens [Ransohoff and Perry, 2009]; these glial changes may not have a major impact on the Cho concentration, because they may not induce changes in membrane turnover. It would be important to improve our understanding of what underlying pathological processes are responsible for Cho signal change in MS. Interestingly, the association between Cho and TWT became stronger when the spatial variation of this metabolite concentration was taken into account, suggesting that attention to spatial variation permits the identification of metabolic changes that are clinically relevant.

In patients with PPMS we also detected a significant association between lower Cr concentration and higher EDSS, which may suggest that a less intact energy metabolism, as reflected by Cr [Wyss and Kaddurah‐Daouk, 2000] has a negative effect on clinical disability. This is in line with the recent hypothesis that energy metabolism, which originates in the mitochondria, plays an important role in the pathogenesis of MS [Di et al., 2010] and in determining disability in MS [Ciccarelli et al., 2010a, 2010b]. Interestingly, greater levels of Cr have also been associated with glial proliferation [Suhy et al., 2000], and we found greater Ins in RRMS than PPMS. Previous studies have reported an association between lower Cr concentration in the cortical GM and greater disability in patients with RRMS [Chard et al., 2002], and we have now extended these results to the CST and patients with PPMS. In addition, in patients with RRMS, we reported a significant, albeit modest, association between greater Ins concentration and greater disability, as measured by the TWT, suggesting that gliosis may be a pathological process of clinical relevance in the relapsing forms of MS [Kirov et al., 2009].

Although we hypothesized that patients with PPMS would show lower NAA than RRMS and healthy controls, because of histological findings [Tallantyre et al., 2009], and previous imaging investigations [Chard et al., 2002; Ciccarelli et al., 2010a, 2010b, 2007; Sastre‐Garriga et al., 2005; Suhy et al., 2000], we did not detect significant differences in tNAA levels between groups. This may due to the fact that we used a different CSI protocol and analysis, and included MS patients with different characteristics. Additionally, we found a relatively large variability in tNAA concentration that could have contributed to the lack of tNAA concentration differences in terms of tNAA differences between groups, because it was much larger than the difference in tNAA between groups (10% vs. 5%). The observed SDs associated with mean tNAA concentrations were indeed higher than those previously reported by other authors [Chard et al., 2002; Sastre‐Garriga et al., 2005]. This variability in tNAA concentration could be due to the fact that we entered in the statistical analysis all the available voxels obtained along the CST (i.e., in different regions of the tract) at the same time. Our analysis has demonstrated that the tNAA concentration increased from the inferior voxels towards the corona radiata, even when correcting for the WM fraction, which also changed across the CST. Therefore, this variability in tNAA levels can be reduced by grouping voxels located in the same region within a tract (as shown in the Supporting Information Table 1). Additionally, we performed a sample size calculation to estimate the number of subjects needed to detect (with 80% power at 5% significance) a difference in tNAA concentration of 4–5% (as observed in our study) between patients and controls, using the means and SDs of tNAA concentrations provided by our data, and standard methods for comparisons of means [Armitage et al., 2002]. We found that relatively large sample sizes would be required to detect significant tNAA differences (68 RRMS and 68 controls; over 98 PPMS patients and 98 controls). The metabolite‐specific reliability of fit can be guaranteed by selecting only those metabolites that fall below the 20% Cramer‐Rao Lower Bound (CRLB) upper limit (see Supporting Information Table 2). From a methodological point of view, an increase in TE might increase the chance of finding significant differences in tNAA levels between groups, since a previous meta‐analysis has shown that studies with TE < 90 ms tended to show no significant differences in tNAA levels between MS patients and controls [Caramanos et al., 2005]. Potential improvements in the technique that would lead to a better quantification of tNAA and include a higher field strength (3 T), better shimming and a greater acquisition matrix size to reduce the point spread function (PSF), may also contribute to reduce the variability in tNAA concentration.

A technical limitation of CSI studies is that the PSF contributes to the definition of the voxel size and should be taken into account by evaluating the contamination to the voxel of interest originating from voxels at neighboring positions. In particular, it is known that the limited sampling size of the CSI grid has a broadening effect on the PSF, which means that the actual voxel size measured at the FWHM may be larger than the prescribed one [Jansen et al., 2006], and that the prescribed voxel is therefore contributing to less than 100% of the measured signal at each voxel position. In this study we examined the prescribed voxel, without evaluating the effect of the PSF, and it is therefore possible that partial volume effects from neighboring voxels may have contributed to the observed metabolite concentrations. Future CSI studies should consider the possibility of improving the PSF during data acquisition or postprocessing.

In addition, our statistical approach was very conservative, and adjusted for several variables, and the number of patients in each group was relatively low. A possible limitation of this study is that voxels may contain a very small percentage of GM (or lesional tissue) in addition to WM, which is unavoidable when performing CSI. To correct for the possible contribution to the metabolite concentrations originating of different amounts of tissues in each voxel, we corrected all our statistical analysis for the tissue segment fractions calculated within each voxel.

In conclusion, the application of CSI to coronal‐oblique slices provides insights into the pathological processes in the CST that are related to disability in RRMS and PPMS. Our results also confirm that taking account of the spatial variation that may exist in the concentration of the metabolites along WM tracts [Goldsmith et al., 2011] improves the ability to detect differences in Cr between patients and controls and significant correlations between Cho and clinical disability. From a technical point of view, we employed an innovative CSI methodology that could be useful in future studies in MS or other neurological disease to quantify metabolite concentrations along the main WM tracts in the brain, and formulate location‐specific hypotheses.

## Supporting information

Supporting Information Table 1.

Supporting Information Table 2.
