# Supplementary material for: Spatial variability and changes of metabolite concentrations in the cortico‐spinal tract in multiple sclerosis using coronal CSI
Source: Hum Brain Mapp. 2012 Dec 26;35(3):993–1003. doi: 10.1002/hbm.22229 (PMC4238834; doi:10.1002/hbm.22229)
Supplement: Supplementary file 1 — Supporting Information Table 1. [file HBM-35-993-s001.doc]

**Supplemental table 1. tNAA concentrations**

|  | **tNAA concentration** | | |
| --- | --- | --- | --- |
|  | **Controls**  tNAA mean (SD)  *Number of voxels (N)* | **RRMS**  tNAA mean (SD)  *Number of voxels (N)* | **PPMS**  tNAA mean (SD)  *Number of voxels (N)* |
| **Voxel position within the CST** |  |  |  |
| **Cerebral Peduncle** | 8.352 (2.584)  *N=23* | 8.534 (1.368)  *N=21* | 8.534 (1.368)  *N=22* |
| **Above Cerebral Peduncle** | 10.607 (1.332)  *N=32* | 9.706 (1.401)  *N=24* | 9.331 (1.040)  *N=26* |
| **Internal Capsule** | 10.811 (1.870)  *N=32* | 10.481 (1.843)  *N=26* | 10.454 (1.397)  *N=26* |
| **Above Internal Capsule** | 11.125 (1.740)  *N=32* | 11.876 (1.317)  *N=25* | 11.547 (1.535)  *N=26* |
| **Corona Radiata** | 11.732 (1.387)  *N=32* | 10.608 (1.385)  *N=25* | 10.656 (1.506)  *N=26* |
| **After all voxels along the CST have been averaged within each subject^a^** | 10.658 (1.186)  *N=151* | 10.286 (0.746)  *N=121* | 10.105 (0.919)  *N=126* |
| **All available voxels along the CST (without intra-subjects averaging)^b^** | 10.640 (2.340)  *N=151* | 10.215 (2.146)  *N=121* | 10.105 (2.142)  *N=126* |

**Supplemental table 1 (footnote).**

The values reported in this table are obtained by segmenting the CST in 5 regions and averaging each metabolite concentrations within each region within controls, RRMS patients and PPMS patients. **a:** These values are also shown in Table 2, and represent the mean value of tNAA within each subject, regardless of voxel position along the CST; **b:** These values were obtained by averaging tNAA levels of all available voxels along the CST (without performing the intra-subject averaging).

Note that when the mean values of tNAA are obtained using tNAA levels from all the available voxels, the associated SD is larger than when perform the intra-subject averaging. In addition, when we group voxels located within the same region of the tract, the SDs of tNAA reduce when compared to the SDs of tNAA of all available voxels.

*Abbreviations:* SD: standard deviation; tNAA: N-acetylaspartate and N-acetlylaspartylglutamate; RRMS: relapsing-remitting multiple sclerosis; PPMS: primary progressive multiple sclerosis.
