# Supplementary material for: Spatial variability and changes of metabolite concentrations in the cortico‐spinal tract in multiple sclerosis using coronal CSI
Source: Hum Brain Mapp. 2012 Dec 26;35(3):993–1003. doi: 10.1002/hbm.22229 (PMC4238834; doi:10.1002/hbm.22229)
Supplement: Supplementary file 2 — Supporting Information Table 2. [file HBM-35-993-s002.doc]

**Supplemental table 2. Cramer-Rao Lower Bound (CRLB) values for each metabolite**

| **Metabolites** | **Mean (SD) %**  **(Range: minimum, maximum)** | | |
| --- | --- | --- | --- |
|  | **Controls** | **RRMS** | **PPMS** |
| **tNAA** | 9.212 (2.670)  (5, 18) | 10.091 (2.683)  (5, 18) | 9.452 (2.535)  (6, 18) |
| **Cho** | 14.237 (2.534)  (9, 19) | 14.608 (2.685)  (7, 19) | 14.918 (2.747)  (9, 19) |
| **Cr** | 13.664 (2.489)  (9, 19) | 14.109 (2.465)  (7, 19) | 13.640 (2.324)  (9, 19) |
| **Ins** | 15.234 (2.422)  (10, 19) | 15.136 (2.757)  (8, 19) | 15.653 (2.779)  (9, 19) |

**Supplemental table 2 (footnote).**

Average of the CRLB values per metabolite across the subjects. The range of CRLBs are also reported.

*Abbreviations:* SD: standard deviation; tNAA: N-acetylaspartate and N-acetlylaspartylglutamate; Cho: choline-containing compounds; Cr: creatine and phosphocreatine; Ins: myo-inositol; RRMS: relapsing-remitting multiple sclerosis; PPMS: primary progressive multiple sclerosis.
